# Kinetics and Potential Mechanisms of LDPE and PBAT Microplastics Biodeterioration by Soil Bacteria *Bacillus cereus* L6

**DOI:** 10.3390/microorganisms14010179

**Published:** 2026-01-14

**Authors:** Jiayang Hu, Tianyu Liu, Jinpeng Zhang, Yong Yu, Jincai Ma, Yanjun Li

**Affiliations:** 1 Key Laboratory of Ground Water Resource and Environment, Ministry of Education, Jilin University, Changchun 130021, China; hjy23@mails.jlu.edu.cn (J.H.); jincaima@jlu.edu.cn (J.M.); 2 Jilin Provincial Key Laboratory of Environmental Ecology in Black Soils, Research Center of Regional Development and Environment, Northeast Institute of Geography and Agroecology, Chinese Academy of Sciences, Changchun 130102, China; liutianyuu@mails.jlau.edu.cn (T.L.); zhangjinpeng@iga.ac.cn (J.Z.); yuyong@iga.ac.cn (Y.Y.); 3 College of Horticulture, Jilin Agricultural University, Changchun 130118, China

**Keywords:** biodeterioration, surface characterization, FTIR, XRD, thermal stability, genome sequencing

## Abstract

Low-density polyethylene (LDPE) and poly (butylene adipate-co-terephthalate) (PBAT) agricultural films are major components of microplastics (MPs) and their contamination in agriculture due to their difficulty to recycle. However, potential degradation mechanisms of MPs from LDPE and PBAT in agricultural soils are still unclear. Here, we isolated a strain of *Bacillus cereus* L6 from long-term agricultural MP-contaminated soil and analyzed its potential biochemical pathways involved in LDPE and PBAT turnover through functional prediction from shotgun genome sequencing. After 28 days of incubation with MPs, *Bacillus cereus* L6 caused a net mass loss of 0.99% LDPE-MPs/28 days and 3.58% PBAT-MPs/28 days. The surfaces of LDPE and PBAT degraded in bioassays added with *Bacillus cereus* L6 showed wrinkles, cracks, and pits, accompanied by an increase in roughness. The crystallinity and thermal stability of both LDPE- and PBAT-MPs were decreased and the hydrophobicity of PBAT-MPs was reduced. Whole-genome sequencing analysis showed that *Bacillus cereus* L6 potentially encoded genes for enzymes related to the biodeterioration of additives in LDPE and PBAT. Moreover, genomic CAZymes predictive analysis showed that genes related to oxygenases and lyases were annotated in the strain L6 Auxiliary Activities family. These findings offer a theoretical foundation for deeper exploration into the degradation and metabolic processes of MPs from discarded agricultural plastics in the environment.

## 1. Introduction

Agricultural plastic films (APFs) perform an essential role in ensuring food production, enhancing the efficiency of irrigation, preventing pests and diseases, and increasing temperature and humidity, and so on [1]. It is estimated that the use of APFs has reached 1.3 million tons in 2021 from China. The area of crops covered by APFs could reach 0.17 billion hectares [2]. Low-density polyethylene (LDPE) is extensively applied to agriculture [3]. However, it is difficult to degrade in the natural environment due to its strong chemical stability [4]. Poly (butylene adipate-co-terephthalate) (PBAT) has been utilized extensively as a biodegradable film because it also exhibits strong toughness and ductility [5]. Frequent use of APFs can transform them into microplastics (MPs) by mechanical friction, UV irradiation, and biodegradation [6]. It is estimated that approximately 100 trillion particles of MPs enter agricultural soils in China due to the extensive use of APFs [7].

MPs can have a negative impact on soil ecosystems [8], as they are difficult to recover once retained in the soil and their natural degradation may take hundreds of years [9]. Therefore, it is important to develop an environmentally friendly method, such as microbial degradation, to eliminate or effectively degrade residual MPs in the environment [10]. It has been shown that microbes screened from landfills [11], soils [12], marine environment [13], compost [14], and the intestines of worms [15] are able to degrade MPs. These diverse microbial sources demonstrate that MP-degrading capabilities have evolved across multiple ecological niches exposed to MPs pollution. It has been demonstrated that a *Rhodococcus* A34 strain, extracted from plastic waste, degraded PE-MPs at a rate of 1% after 30 days of incubation at 30 °C [16]. Evidence also indicates that a strain of *Bacillus* from a plastic landfill that was able to biodegrade LDPE film, with incubation at 25 °C for 4 months resulting in a 43% reduction in the mass of the LDPE film [10]. Furthermore, a *Roseibium aggregatum* ZY-1 from seawater degraded PBAT films by 2.29% when incubated for 20 days [17]. The distinct degradation behaviors of these plastics stem from their molecular structures. The active ester bonds in PBAT make it susceptible to hydrolysis or enzymatic cleavage, enabling relatively faster degradation [18,19]. In contrast, PE, composed of inert C–C single bonds, lacks reactive sites and primarily undergoes slow physical fragmentation and oxidation [20].

Potential mechanisms for the microbial degradation of MPs mainly include a combination of enzyme-catalyzed reactions, including hydrolysis, oxidation, reduction, and esterification [21]. These reactions catalyze a series of reactions that contribute to polymer bond breaking or polymer functional group modification. Two strains of *Pseudomonas* spp. were isolated from sewage water that degrade PE APF, and whole-genome analysis has shown that these two degrading bacteria are involved in the coding of enzymes related to plastic degradation, such as monooxygenases, dioxygenases, and hydroxylases, that catalyze a series of oxidation–reduction reactions [22]. Moreover, bacterial plasmids carry genetic information that can encode specialized proteins that enable the bacteria to metabolize specific pollutants [23]. However, studies on the mechanisms of microbial degradation of MPs are not yet comprehensive. Current research gaps highlight the need for more systematic investigations using advanced genomic approaches. Therefore, it is of great significance to focus on bacterial plasmids to study their degradation mechanisms using shotgun DNA sequencing.

In this study, we hypothesized that the long-term selection pressure of APF contamination would enrich the native soil microbiome for bacteria possessing a broader and more efficient metabolic capacity to degrade both conventional (e.g., LDPE) and biodegradable (e.g., PBAT) plastic polymers. The objectives of this study were to (1) isolate and identify bacterial strains with the ability to degrade both LDPE and PBAT rapidly; (2) characterize the biodeterioration effects on LDPE- and PBAT-MPs; and (3) elucidate the potential microbial degradation mechanisms of MPs through shotgun sequencing. This research may contribute to understanding the degradation mechanism of MPs and contribute to the mitigation of environmental pollution by agricultural film-derived MPs.

## 2. Materials and Methods

### 2.1. Preparation of Microplastics

LDPE and PBAT APF were purchased from Jialemi Agro Plant (Changchun, China). The LDPE and PBAT film were cut into irregular fragments with approximate dimensions of 5 mm using sterile scissors. The fragments were then sieved using standard stainless-steel test sieves. MPs that passed through a 5 mm sieve but were retained on a 3 mm sieve were collected for all subsequent experiments.

The prepared MPs underwent a cleaning and sterilization protocol to serve as a uniform, contaminant-free substrate. They were soaked in 2% SDS, 75% ethanol, and 95% ethanol for 4 h, during which it was cleaned with a numerically controlled ultrasonic cleaner and a vortex mixer with vibration for 30 min, respectively. After soaking, the MPs were rinsed with sterile water three times, and the water on the surface of the MPs was absorbed by sterile filter paper in an ultra-clean bench.

### 2.2. Enrichment, Isolation, and Identification of LDPE- and PBAT-MPs-Degrading Strains

Soil samples for isolation of MP-degrading bacteria were taken from peanut fields covered with long-term agricultural film in Fuyu, China (longitude: 125.81°; latitude: 45.09°). Soil was collected from 30 cm below the agricultural film cover, then 1 kg of soil samples were packed in glass vials and stored in iceboxes to take back to the laboratory [12].

Soil samples were accurately weighed to be 1.0 g, were added to the tube with liquid trace carbon source medium, placed at 180 rpm, and shaken at 35 °C, and then sequentially diluted to the concentration of 10^−4^ according to a gradient and used for the solid plate enrichment of MPs-degrading bacteria. The MPs were spread flat on the solid trace carbon source medium, and 100 μL of soil suspension at a concentration of 10^−4^ was aspirated and coated on the solid medium and incubated at 35 °C for 10 d. MPs solid trace carbon source medium without soil suspension was incubated under the same conditions as a blank control. After 10 days, the MPs were transferred to a new solid trace carbon source medium and underwent a secondary enrichment for another 10 days.

After a total of 20 days of plate enrichment, the MPs from the secondary enrichment plates were washed with sterilized PBS phosphate buffer (pH = 7.2, 0.1 M) with shaking to make bacterial suspension. Then, 100 μL of bacterial suspension was coated on the inorganic salt medium spread with MPs, and incubated for 30 days at 35 °C, and incubated with MP film solid inorganic salt medium that was not coated with bacterial suspension under the same conditions, as a blank control. The solid plates were incubated for 30 d; the colonies visible to the naked eye were picked and streaked on LB medium for purification and used to isolate the bacteria that may have degrading properties. The specific formulations of the liquid trace carbon source medium, solid trace carbon source medium, inorganic salt medium, solid plates, and LB medium required in this study are provided in Text S1. Strain L6 has been determined using 16S rRNA gene sequence analysis [24], and information on primer selection is given in Text S2. The 16S rRNA gene sequence of strain L6 has been submitted to the NCBI GenBank database under the accession numbers CP162629. Phylogenetic analysis was performed using MEGA 11 software.

### 2.3. Biodeterioration of LDPE- and PBAT-MPs

To assess the biodeterioration potential of *Bacillus cereus* L6, a comparative incubation experiment was established. All experiments were conducted in 250 mL Erlenmeyer flasks containing 100 mL of sterile inorganic salt medium (pH = 7.0). Two parallel treatment groups were set up for each type of MPs (LDPE and PBAT, 2 g/L). For the experimental group, the medium supplemented with MPs was inoculated with a 10% (*v*/*v*) active culture of *Bacillus cereus* L6 (OD_600_ ≈ 0.8). For the CK group, the medium was supplemented with MPs but without bacterial inoculation. Prior to incubation, all MPs (for both experimental and control groups) underwent an identical cleaning and sterilization protocol. The detailed procedure is described in Section 2.1. All flasks were incubated at 35 °C and 150 rpm for 28 days. The growth of strain L6 in the experimental group was monitored by measuring the optical density (OD_600_) of the culture broth at 7-day intervals.

After the 28-day incubation, MPs from all Erlenmeyer flasks were retrieved and subjected to the same rigorous cleaning procedure as described in Section 2.1 to remove any adhering biomass, salts, or medium components. The logistic growth model was employed to analyze microbial growth dynamics on different MPs. The model is described by the following equation:

OD_600_(t) = A/[1 + exp (−k (t − t_0_))]

OD_600_(t) is the optical density at time t, A is the maximum OD_600_ (carrying capacity), k is the growth rate constant (day^−1^), and t_0_ is the inflection point time (day) corresponding to the point of maximum growth rate.

The weight loss of LDPE- and PBAT-MPs was then determined according to the following formula:

Weight loss (%) = (W_D28_ − W_D0_)/W_D0_ × 100%

W_D0_ is the mass for LDPE- or PBAT-MPs. W_D28_ is the mass for LDPE- or PBAT-MPs following 28 d of incubation with strain.

### 2.4. Characteristics of LDPE- and PBAT-MPs

Changes in the surface morphology of LDPE- or PBAT-MPs were observed by scanning electron microscope (SEM, JSM-IT500, JEOL Ltd., Tokyo, Japan). Energy-dispersive X-ray spectroscopy (EDS) was conducted along with SEM analysis. The surface erosion for the control and L6-degraded LDPE- or PBAT-MPs outer layers was investigated by an atomic force microscope (AFM, alpha 300 A, WITEC, Ulm, Germany). A contact angle analyzer (OCA 15EC, DataPhysics Instruments GmbH, Filderstadt, Germany) was used to assess the surface hydrophobicity of LDPE- or PBAT-MPs. Fourier Transform Infrared Spectroscopy (FTIR, Nicolet iS5, Thermo Fisher Scientific, Waltham, MA, USA) was applied to analyze functional groups for LDPE- and PBAT-MPs. X-ray diffraction (XRD, D8 Advance, Bruker AXS GmbH, Karlsruhe, Germany) was employed to determine the change in crystallinity for LDPE- and PBAT-MPs. The thermal stability of the control and LDPE- and PBAT-MPs treated with L6 was analyzed using a thermogravimetric analyzer (TGA 4000, PerkinElmer, Inc., Waltham, MA, USA). For more information on the characterization of MPs, see Text S3.

### 2.5. Shotgun Sequencing and Annotation

The genomic DNA of strain L6 (including extrachromosomal elements) was first extracted, followed by the construction of SMRT Bell library using the SMRT kit (Pacific Biosciences, Menlo Park, CA, USA). The constructed library was quantified by Qubit, and the insert size was detected by Agilent 2100 (Agilent Technologies, Santa Clara, CA, USA), and finally sequenced by PacBio platform to an average depth of 100×. Using NEBNext kit (New England Biolabs, Ipswich, MA, USA), the whole library preparation was completed by end repair, the addition of A-tail, the addition of sequencing junction, purification, and PCR amplification. After the library construction was completed, Qubit 2.0 was used for preliminary quantification, and Q-PCR was used to accurately quantify the effective concentration of the library to ensure the quality of the library. Detailed methods are described in the Text S4.

The resulting sequencing reads were processed using a dedicated bioinformatic pipeline. Low-quality reads were filtered using SMRT Link v8.0. De novo assembly was performed with Canu (version 2.0) to generate a single, gapless contig. Gene annotation was conducted by predicting coding sequences with GeneMarkS v4.17 and performing BLASTP searches (E-value < 1 × 10 ^−5^, minimal alignment length > 40%) against multiple databases, including NR, Swiss-Prot, KEGG, COG, and GO. Genomic synteny analysis was carried out by aligning the assembled genome to reference genomes using the MUMmer v4.0.0 and LASTZ tools v1.04.00.

### 2.6. Statistical Analysis

SPSS Statistics (version 25.0; IBM Corp., Armonk, NY, USA) and Originpro (version 2023; OriginLab Corp., Northampton, MA, USA) software were used to statistically analyze and graphically present the data. One-way ANOVA was used to analyze the changes in the degradation rate of MPs in the different treatment groups. Repeated measures ANOVA tests were conducted to assess different MPs groups’ effect on the growth of the strains with time as the repeated measures. When the data violated the Mauchly’s test, the Greenhouse–Geisel correction was employed to report the results [25]. Post hoc pairwise comparisons were carried out using Tukey’s honestly significant difference (HSD) test to determine specific differences between groups. All statistical tests were considered significant at *p* < 0.05.

## 3. Results and Discussion

### 3.1. Isolation and Identification of LDPE and PBAT-MPs Potential Degrading Bacteria

**Figure 1:**
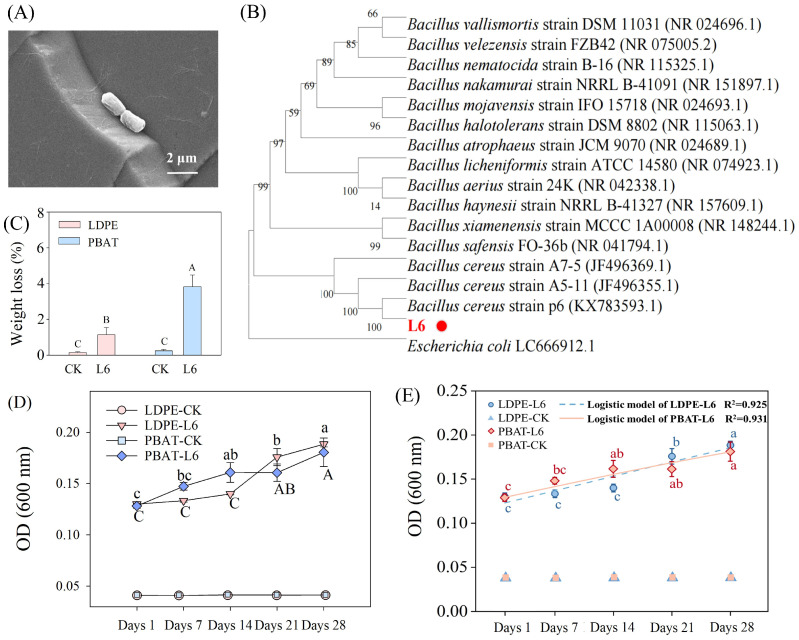
Morphology of strain L6 (**A**). Phylogenetic tree of strain L6 constructed by MEGA based on the neighbor-joining method. The numbers on each clade are bootstrap values (**B**). Strain L6 is highlighted with a red dot. The phylogenetic tree was constructed using the neighbor-joining method based on Kimura two-parameter distances. Bootstrap values (1000 replicates) are shown at the nodes. Weight loss of LAPE- and PBAT-MPs after biodeterioration by strain L6 (**C**); different capital letters indicate differences between treatments. CK: the control group without strain L6. Changes in OD values of strain L6 during 28 days of co-culturing MPs with strain L6; different lowercase letters indicate changes in the PBAT-MPs group at different times, and different capital letters indicate changes in the LDPE-MPs group at different times (**D**). Growth kinetics of strain L6 on LDPE-MPs and PBAT-MPs fitted with the logistic growth model (**E**).

The strain L6 was isolated from the peanut farm soil that had been mulched for a long time with agricultural film it was able to use LDPE- and PBAT-MPs as its sole carbon source. The persistent presence of MPs in soil likely creates an ecological niche favorable to microorganisms with MPs degradation capabilities, promoting adaptive evolution in strain L6. As shown in Figure 1A, the morphology of strain L6 was rod-shaped cells. The results of 16S rDNA gene sequencing were matched against NCBI database with the help of the BLAST tool v2.2.26. MEGA 11 software was employed to construct the phylogenetic tree. Srain L6 was genetically the most closely related to accession KX783593.1; therefore, strain L6 was considered to be *Bacillus cereus* (Figure 1B). The sequence of strain L6 was uploaded to NCBI GenBank database (Accession number PQ047533).

### 3.2. Biodeterioration of LDPE- and PBAT-MPs by Strain L6

To assess the degradation ability of strain L6, biodegradation tests were performed using commercial LDPE and PBAT agricultural film MPs. The degradation rate was 1.14% LDPE-MPs/28 days and 3.82% PBAT-MPs/28 days. After subtracting the background loss observed in the control group (0.15% for LDPE-MPs and 0.24% for PBAT-MPs, representing physical losses during recovery, washing, and weighing), the net biodegradation rates attributable to strain L6 were calculated as 0.99% for LDPE-MPs and 3.58% for PBAT-MPs (Table S1, Figure 1C). This is comparable to the degradation efficiency reported in previous studies, where single bacterial strains isolated from pelagic waters or landfill sites were incubated with PE-MPs for 30 days [16,26]. Notably, the slightly higher degradation rate observed for PBAT-MPs (3.58% PBAT-MPs/28 days) aligns with its more biodegradable nature compared to LDPE, which exhibits greater recalcitrance. The degradation rate of PBAT-MPs in our results was also comparable to that of bacterium ZY-1 isolated from seawater in a previous study, which showed a degradation rate of 2.29% after co-culturing with PBAT for 20 days [17]. The observed mass loss may be attributed to biodeterioration-related surface erosion, additive leaching, and structural modification. This suggests that strain L6 possesses the ability to degrade both conventional MPs and biodegradable MPs, although the degradation efficiency varies.

Bacterial growth rates of strain L6 were determined every 7 days during the 28-day incubation period with LDPE-MPs or PBAT-MPs (Figure 1D). To quantify the growth dynamics, a bacterial growth kinetics of strain L6 was analyzed by fitting the data to a logistic growth model [27,28,29]. The model demonstrated an excellent fit to the data for both types of MPs (Figure 1E, Table S2, R^2^ > 0.92). As shown in Table S3 and Figure 1E, strain L6 grew on PBAT-MPs as the carbon source with a short adaptation phase and negligible lag time, according to the logistic model. This immediate growth response, where the average OD_600_ increased from 0.1281 ± 0.0039 to 0.1608 ± 0.0166 in the first 14 days, aligns with PBAT’s biodegradable nature. From day 14 to day 21, the OD of strain L6 stabilized, and then increased to 0.1804 on day 28. In contrast, a pronounced lag phase of approximately 10–14 days was observed for strain L6 grown on LDPE as the sole carbon source. Consistent with this, the model inflection point (t_0_) was estimated at 12.75 days. The initial period (days 1–14) was characterized by a marginal increase in OD_600_ from 0.1300 to 0.1399, followed by a rapid exponential growth phase (days 14–28), where the OD_600_ surged to 0.1883.

At initial stage of biodeterioration, the OD value of L6 in LDPE-MPs treatment was lower than that in PBAT-MPs treatment, probably because PBAT is a biodegradable plastic, whereas LDPE is more difficult to degrade [30]. The strain L6 requires an adaptation period before it can start degrading LDPE-MPs [31]. The OD value in the CK group did not significantly change within 28 days, indicating that the process was free from contamination and confirming that observed growth was indeed dependent on MPs biodeterioration. These controlled experimental conditions provide robust evidence for strain L6’s genuine MP biodeterioration capacity.

### 3.3. Biodeterioration Characteristics of LDPE- and PBAT-MPs by Strain L6

**Figure 2:**
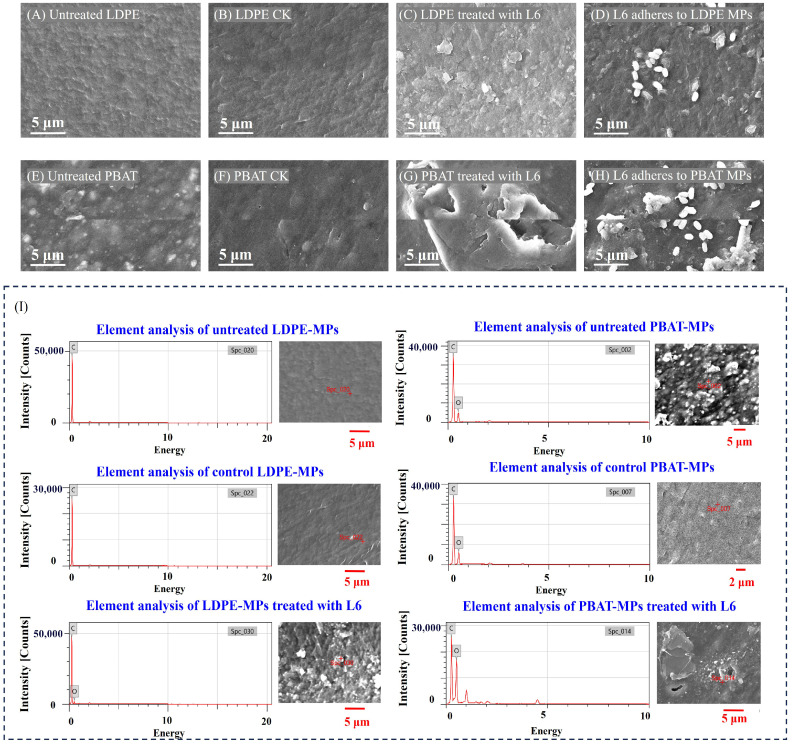
Scanning electron microscope images to analyze the surface morphology of LDPE- and PBAT-MPs. Untreated LDPE- and PBAT-MPs before incubation (**A**,**E**). Control LDPE- and PBAT-MPs were incubated for 28 days without strain L6 (**B**,**F**). LDPE- and PBAT-MPs were incubated in the presence of strain L6 for 28 days, respectively (bacterial cells were removed prior to imaging to evaluate surface morphology of MPs; detailed methods are described in Section 2.1) (**C**,**G**). Strain L6 attached to the surface of LDPE- and PBAT-MPs, respectively (**D**,**H**). All samples were subjected to platinum sputter coating in an argon atmosphere (0.3 MPa) at a current of 25 mA. MPs were taken at a magnification of 5000×. EDS analysis of LDPE- and PBAT-MPs (**I**). The labels C and O in the figure represent the elements carbon and oxygen, respectively.

Folds and protrusions were observed on the surface of pristine LDPE- and PBAT-MPs (Figure 2A,E), and the control LDPE and PBAT showed slight folds and pores after 28 days of oscillation in the medium without L6 (Figure 2B,F), which might be due to mechanical damage caused by continuous agitation during the experimental period. In contrast, the surface of the LDPE-MPs biodeteriorated by strain L6 appeared cracked with detached particles (Figure 2C), while PBAT-MPs showed extensive holes and folds on the surface (Figure 2G). Notably, while the SEM images revealed minor residual structures (Figure 2D,H), these minimal remnants did not compromise the clear visualization of biodeterioration-induced surface alterations. The observed cracks (LDPE) and holes (PBAT) correlated well with the measured weight losses, suggesting active biodeterioration by strain L6 [32,33].

The EDS analysis revealed that the control LDPE-MPs contained 100% carbon by weight and 100% carbon by atom, while the relative weight of carbon and oxygen for LDPE-MPs treated by L6 were 93.37% and 6.63%, respectively (Figure 2I, Table S4). The increase in oxygen content in the biodeterioration samples may suggest strain L6’s capacity to chemically modify MPs surfaces through microbial activity. Similar evidence of surface oxidation has been consistently reported across diverse MP microbe systems, such as in the biodegradation of polyethylene terephthalate (PET) and polystyrene (PS) by *Bacillus cereus* [34], and PE by the marine strain of *Pseudalkalibacillus* sp. [20]. The relative weights of carbon and oxygen in control PBAT-MPs were 79.97% and 20.03%, and the atomic relative weight percentages were 84.17 and 15.83, respectively. In contrast, the weight percentages of carbon and oxygen in the L6-degraded PBAT-MPs were 57.21% and 42.79%, and the atomic weight percentages were 64.04 and 35.96, respectively (Figure 2I, Table S4). The decrease in carbon in both LDPE- and PBAT-MPs suggests that strain L6 was able to utilize LDPE- and PBAT-MPs as its primary and essential source of carbon for growth under the experimental conditions [35]. In addition, it is possible that the oxygen in MPs was increased due to catalytic oxidation–reduction reactions in MPs under the influence of strain L6 secretions [36]. The substantial oxygen incorporation observed particularly in PBAT biodeterioration products highlights the potential for microbial processes to fundamentally alter MPs chemistry.

**Figure 3:**
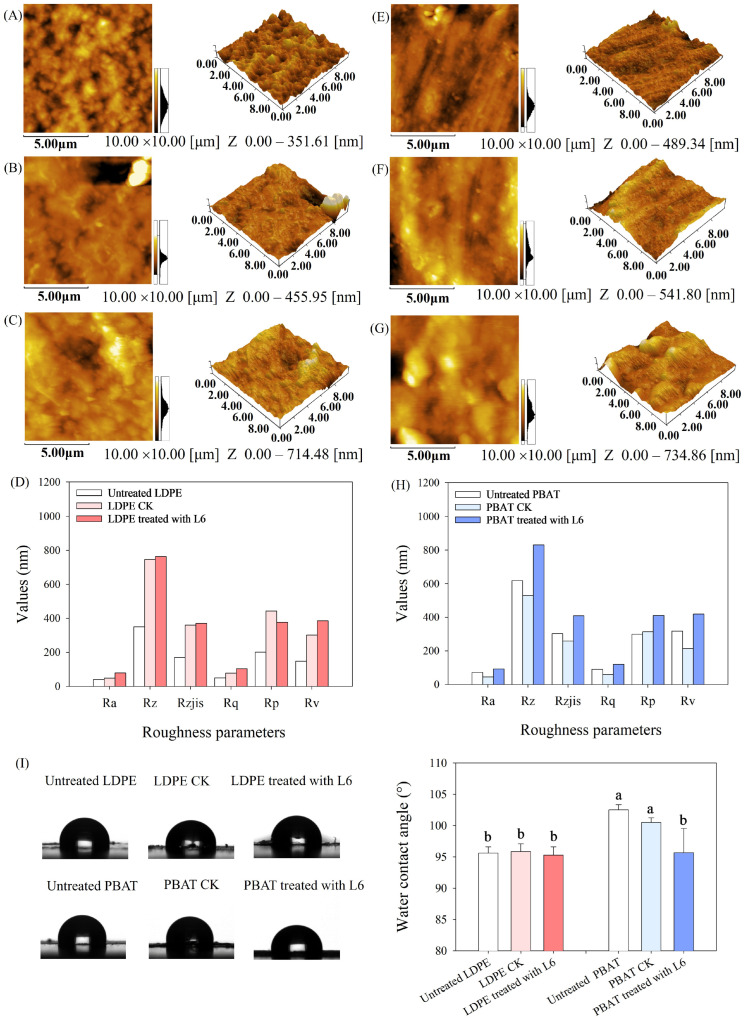
Micrographs of AFM-2D and AFM-3D of pristine LDPE- and PBAT-MPs (**A**,**E**), control LDPE- and PBAT-MPs (**B**,**F**), and LDPE- and PBAT-MPs biodeterioration by strain L6 (**C**,**G**). Changes in roughness-related indexes of LDPE- and PBAT-MPs (**D**,**H**). Changes in water contact angle of LDPE- and PBAT-MPs (**I**). Different lowercase letters indicate changes in the different MPs group. Ra: Arithmetic mean deviation of the assessed profile. Rz: Maximum height of profile. Rzjis: Japanese Industrial Standard version of Rz, calculated as the average difference between the five highest peaks and the five deepest valleys within a sampling length. Rq: Root mean square deviation of the assessed profile. Rp: Maximum profile peak height. Rv: Maximum profile valley depth.

Increased surface roughness was observed for LDPE- and PBAT-MPs biodeterioration induced by strain L6 compared to the control MPs (Figure 3A–C,E–G), as evidenced by the increase in roughness-related parameters (Ra, Rz, Rzjis, Rq, Rp, and Rv, Figure 3D,H). This is consistent with previous studies that bacterial treatment leads to similar morphological changes on polymer surfaces [10,37]. The increase in the surface roughness of LDPE- and PBAT-MPs after incubation in the presence of L6 was attributed to the wrinkles, cracks, and pits on the surface of the MPs caused by the bacteria [38].

No significant change in water contact angles (WCAs) was observed in LDPE-MPs treated with strain L6, whereas the WCAs of PBAT-MPs treated with strain L6 exhibited a significant 6.65% reduction in WCAs relative to pristine PBAT-MPs (Table S1, Figure 3I). This indicates that the hydrophobicity of PBAT-MPs was reduced. The lowered water contact angles demonstrate measurable changes in surface properties that may facilitate enhanced microbial interaction with the MPs substrate. It has been shown that the decrease in the hydrophobicity of MPs can reduce the resistance of MPs to bacteria and thus can increase the degradation of MPs by bacteria [39].

**Figure 4:**
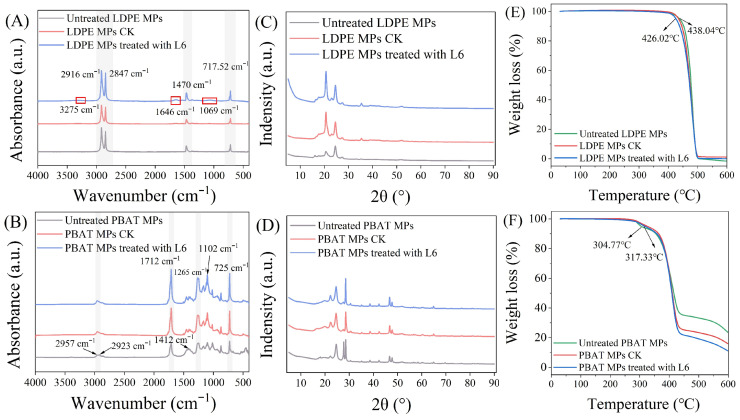
Changes in the FTIR spectra of LDPE- and PBAT-MPs before and after biodeterioration by strain L6 (**A**,**B**). Changes in crystallinity of LDPE- and PBAT-MPs before and after biodeterioration by strain L6 (**C**,**D**). Thermogravimetric analysis of LDPE- and PBAT-MPs before and after biodeterioration by strain L6 (**E**,**F**).

The FTIR spectrum of LDPE-MPs showed characteristic absorption peaks at 2916, 2847, 1470, and 717 waves cm^−1^, which corresponded to PE-MPs [16]. The stretching vibration of –O–H intermolecular hydrogen bonding at 3275 waves cm^−1^, stretching vibration of carbonyl group at 1646 waves cm^−1^, and C–O stretching vibration at 1069 waves cm^−1^ were observed for LDPE-MPs degraded by L6 (Figure 4A), suggesting that the degraded LDPE-MPs incorporated oxygen. Moreover, the carbonyl index was calculated as the ratio of the area for the emerging carbonyl peak (~1646 cm^−1^) to that of the stable aliphatic CH_2_ reference peak (2916 cm^−1^) [40]. This index increased 0.069 ± 0.002 after L6 treatment, demonstrating oxygen incorporation into the polymer, consistent with the EDS results (Figure 2I, Table S4). PBAT-MPs peaked at 2957, 2923, 1712, 1412, 1265, 1102, and 725 waves cm^−1^, corresponding to PBAT in the previous study [18]. The symmetric telescopic vibration peaks for C=O and benzene ring in the main chain at 1712 and 1412 waves cm^−1^ underwent a slight shift displacement after the biodeterioration of the PBAT-MPs by L6 (Figure 4B), suggesting structural modifications in the ester linkages and aromatic components of the polymer backbone. The ratio of the integrated area of the ester carbonyl peak (1712 cm^−1^) to that of the aliphatic CH_2_ peak (2923 cm^−1^, used as an internal reference) was decreased by 5.19 ± 0.87%, indicating partial cleavage of the ester linkages [41,42]. To more precisely quantify the extent of backbone scission, future studies may employ gel permeation chromatography (GPC) analysis to monitor changes in molecular weight, thereby providing a more comprehensive understanding of the biodeterioration mechanism.

As shown in Figure 4C,D, the crystallinity of MPs from the CK group was slightly reduced relative to the original LDPE- and PBAT-MPs. The crystallinity of both LDPE- and PBAT-MPs treated with strain L6 was reduced compared to the CK MPs. This result suggests that strain L6 may use LDPE and PBAT as its primary and essential source of carbon for growth, secrete extracellular enzymes, and catalyze redox reactions after attaching to the surface of MPs, which ultimately leads to changes in the molecular conformation of MPs, and consequently to changes in the degree of crystallinity [43]. This is in line with previous findings that marine microalgae-treated LDPE also showed reduced crystallinity [44], suggesting this may represent a common feature in biological biodeterioration of MPs.

The thermal stability of MPs was analyzed by thermogravimetric analysis (TGA). The temperature of the pristine LDPE-MPs with a 5% weight loss (W5) was 438.04 °C, and the LDPE-MPs were degraded by L6 for 28 days, and the temperature of W5 decreased to 426.02 °C (Figure 4E). For PBAT-MPs, the temperature of W5 after L6 biodeterioration was 317.33 °C, which was higher than the original PBAT-MPs (304.77 °C). The initial thermal stability enhancement suggests complex surface interactions between microbial metabolites and PBAT components during early biodeterioration stages. This may be because antioxidants (such as hindered phenols), which are added during the commercial production of PBAT, become enriched on the surface of PBAT-MPs [45]. These additives are designed to prevent thermo-oxidative degradation during processing, thereby enhancing the thermal stability of the material in the initial stages of thermogravimetric analysis [46]. When the temperature reached 600 °C, the weight loss for the original PBAT-MPs was 76.63%, whereas the weight loss for the PBAT-MPs after undergoing L6 biodeterioration was 89.05% (Figure 4F). The thermal stability of the L6-treated PBAT-MPs eventually decreased with increasing temperature. The results suggested that thermal stability of both the degraded LDPE- and PBAT-MPs was reduced, as evidenced by the reduced crystallinity of the MPs. This is consistent with previous findings that the thermal stability of biodegraded MPs decreased and was accompanied by changes in crystallinity [47]. The biodeterioration of LDPE and PBAT-MPs by strain L6 was further supported by the decrease in thermal stability.

Based on comprehensive analyses using SEM, AFM, WCAs, EDS, FTIR, XRD, and TGA, this study demonstrates the capability of strain L6 to biodeterioration both LDPE- and PBAT-MPs, albeit through distinct mechanisms. For inert LDPE, biodeterioration occurs mainly via surface oxidation and physical erosion, ultimately reducing its crystallinity and thermal stability. In contrast, for biodegradable PBAT, strain L6 not only significantly alters surface morphology and hydrophilicity but also cleaves ester bonds in the polymer backbone, leading to substantial changes in elemental composition. This difference arises from their distinct chemical structures: LDPE’s stable C–C backbone resists breakdown [20], whereas PBAT’s ester bonds offer vulnerable sites for enzymatic attack [18,19]. The consistent evidence from comprehensive analytical techniques demonstrates the potential biodegradation capability of strain L6, supporting its potential application in addressing complex plastic pollution.

### 3.4. Mechanism of LDPE and PBAT Biodeterioration by Strain L6

**Figure 5:**
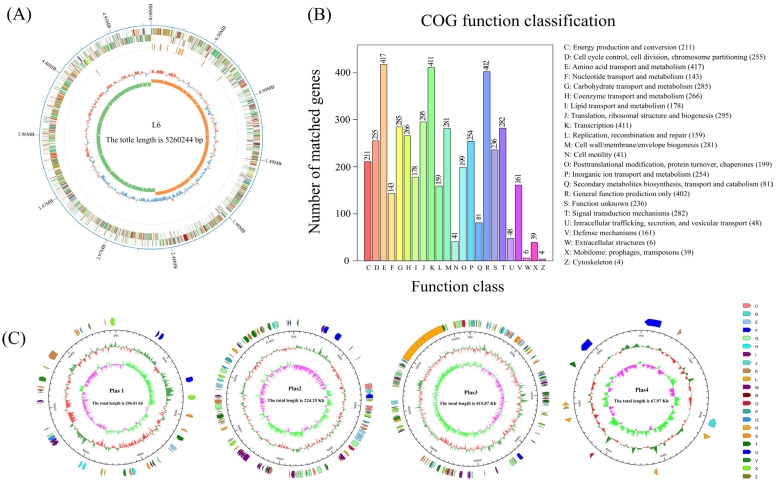
Genomic features of *Bacillus cereus* strain L6. Circular map of the chromosome (**A**). From outer to inner, they are respectively: 1. Genomic sequence position coordinates; 2. Gene functional annotation results; 3. ncRNA and genomic GC content: the inward blue regions indicate areas where the GC content is lower than the genome-wide average, while the outward red regions indicate the opposite, with higher peaks representing greater deviations from the average GC content; 4. Genomic GC skew (G-C/G+C), with inward green regions indicating areas where the G content is lower than the C content, and outward orange regions indicating the opposite. The complete chromosome sequence is available in GenBank under accession number CP162629. Functional classification of predicted CDSs based on the Clusters of Orthologous Groups (COG) database (**B**). Circular maps of the four plasmids identified in strain L6 (**C**). From outer to inner, they are respectively: 1. COG functional annotation classified genes (clockwise arrows indicate genes encoded on the positive strand); 2. Genomic sequence position coordinates; 3. Genomic GC content: inward red regions indicate areas where the GC content is lower than the genome-wide average, while outward green regions indicate the opposite, with higher peaks representing greater deviations from the average GC content; 4. Genomic GC skew: inward purple regions indicate areas where the G content is lower than the C content, while outward green regions indicate the opposite. The plasmid sequences are deposited in GenBank under accession numbers CP162625 (plasmid 1), CP162626 (plasmid 2), CP162627 (plasmid 3), and CP162628 (plasmid 4).

**Figure 6:**
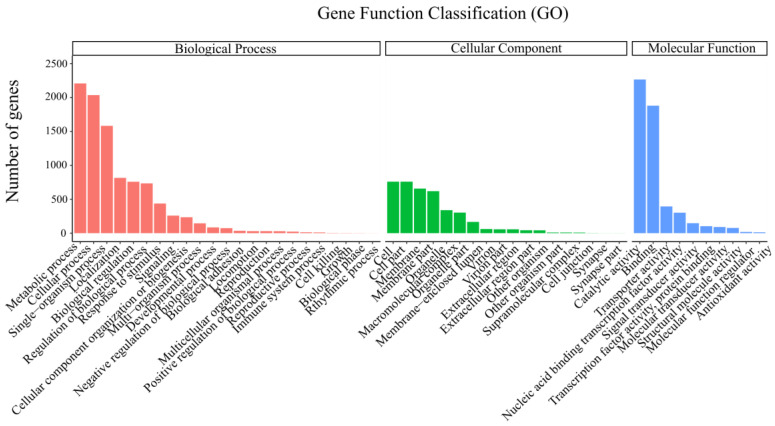
Distribution and number of functionally annotated genes of strain L6 against the GO database. The different colors represent the three primary GO categories: red for Biological Process, green for Cellular Component, and blue for Molecular Function. The bar height indicates the number of genes annotated in each subcategory.

Genomic information helps to hypothesize the plausible mechanisms of the bacterial biodeterioration of MPs [48]. Shotgun sequencing analysis of strain L6 was conducted to reveal the potential set of genes which may be associated with the biodeterioration of MPs. The complete genome sequence of *Bacillus cereus* L6 has been deposited in GenBank under the accession numbers CP162629 (chromosome), CP162625–CP162628 (plasmids). The genome size for *Bacillus cereus* L6 was 5,260,244 bp, which contained 6343 coding sequences (CDSs) and 106 tRNA genes, and the average GC content was 35.26% (Figure 5A). Four additional plasmids were also annotated, and their genome sizes were 206.81 Kb, 224.25 Kb, 415.87 Kb, and 67.97 Kb, respectively (Figure 5B), potentially harboring accessory genetic elements that may contribute to environmental adaptation and pollutant degradation. Functional annotation of the genome of strain L6 using the COG database identified and determined 23 specific functions and number of genes (Figure 5C). Among these, the numbers of CDSs for amino acid transport metabolism and transcription were high, at 417 and 411, respectively, accounting for 8.96% and 8.83% of the total CDSs. The high proportion of genes involved in amino acid metabolism and transcription highlights the strain’s robust protein synthesis machinery, which is fundamental for producing biodeterioration-related proteins. In addition, CDSs of carbohydrate transport and metabolism, coenzyme transport and metabolism, and lipid transport and metabolism accounted for 6.12%, 5.71%, and 3.82% of the total CDSs, respectively. This indicates that the genome of strain L6 includes functional genes related to amino acid, carbohydrate, and lipid metabolism-related functions, which are conducive to providing more energy for degrading pollutants [49]. According to the results of the three major classifications of the Gene Ontology (GO) classification, the genome of strain L6 was annotated with 9547, 3895, and 5289 relevant genes in biological processes, cellular components, and molecular functions, respectively (Figure 6). The functional genes related to metabolic processes accounted for 23.12% of the cellular processes, and the genes related to catalytic activity accounted for 42.82% of the molecular functions. The enrichment of genes related to catalytic and metabolic processes in the genome of strain L6 implies its potential capacity for growth and pollutant degradation [48].

Three pathways associated with biofilm formation were annotated in strain L6 as map02026, map02025, and map05111, enriched for six, two, and ten genes, respectively (Table S3), suggesting that strain L6 also possesses the potential for biofilm formation. Previous studies have shown that a PE-degrading strain, *Pseudomonas aeruginosa*, can attach to MPs and form biofilms [22]. The CAZymes database’s taxonomic annotation results derived the number of genes for oxidoreductases (AAs) as five. In addition, laccase and monooxygenases were annotated in the AAs family (Figure S1, Table S5). Laccase and oxygenases have been described to perform essential roles in the microbial enzymatic degradation of MPs [50]. Bacteria can produce large amounts of laccase for the oxidation of phenolic compounds, which in turn affects the degradation for polystyrene and polyurethane [50]. Oxygenase is a catalyst to coordinate the oxidation of PE plastics and is a major factor in the degradation of PE films [22]. This suggests that strain L6 may attach to the surface of MPs, form biofilms, and subsequently secrete laccases and oxygenases to further biodeterioration of the LDPE- and PBAT-MPs. The combined action of biofilm-mediated surface modification and enzymatic oxidation likely enables the efficient biodeterioration of MPs.

**Figure 7:**
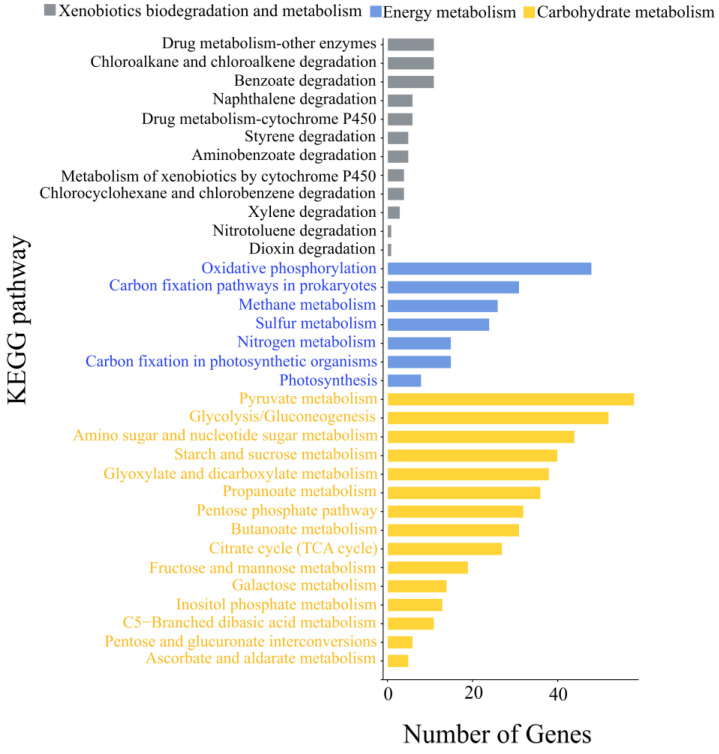
Gene function annotation KEGG metabolic pathway classification map.

A total of thirteen pathways related to biodegradation and metabolism of xenobiotics, seven pathways related to energy metabolism, and fifteen pathways related to carbohydrate metabolism were identified in strain L6 (Figure 7). Notably, in the styrene degradation pathway (map00643), the genes L6_GM002044 and L6_GM002722 were found to be able to encode the enzyme EC3.5.1.4 (Table S6, Figure S2), which possesses the ability to catabolize acrylamide. The presence of these specific genetic determinants suggests strain L6’s potential capacity to biodeteriorate MPs through specialized metabolic routes. Strain L6 was also found to contain a gene (L6_GM005190) for an enzyme associated with ethylbenzene degradation (map00642; EC2.3.1.16, Table S6, Figure S3). The genomic evidence for degradation pathways targeting multiple MPs additives provides a molecular basis for understanding strain L6’s observed MP biodeterioration capabilities. Both acrylamide and ethylbenzene have been shown to be plastic additives in PE plastics [51,52,53]. Six genes associated with the degradation of the plastic additives xylene and limonene (map00622 and map00903) were also found in strain L6, which were L6_GM003690, L6_GM004562, L6_GM005543, L6_GM001485, L6_GM002962, and L6_GM003713 (Table S6, Figures S4 and S5). This suggests that strain L6 may contribute to the removal of PE plastic additives by regulating genes associated with plastic additive-degrading enzymes, ultimately leading to weight loss in LDPE-MPs. It is worth noting that plasmid 2 was found to encode a gene (L6_GM005698) for an enzyme associated with naphthalene degradation (Table S6, Figure S6). Naphthalene has been shown to be an organic additive in PBAT biodegradable APF [45,54]. This is consistent with previous findings that genes involved in pollutant biodegradation are usually contained on a wide range of host plasmids [55,56]. Plasmids are transmissible and can be spread among soil microorganisms through transformation and transduction [57,58], which will contribute to the biodeterioration of MPs. This suggests that strain L6 may act on MPs to achieve biodeterioration by encoding genes for enzymes related to the degradation of plastic additives, expressing the enzymes and secreting them outside of the bacterial body via the bacterial secretion system (map030070).

### 3.5. Limitations

In this study, a bacterial strain capable of deteriorating LDPE- and PBAT-MPs was isolated, characterized, and explored for its mechanism of action. Although the strong concordance between MPs-specific chemical damage and the presence of corresponding catabolic genes in the genome suggests an enzymatic degradation mechanism, direct in vitro validation using purified enzymes remains to be performed. Future validation studies will focus on elucidating the functions of the following key enzymes through gene knockout and heterologous expression studies: the laccases (AA1 family) and monooxygenases (AA3 family) potentially responsible for initiating oxidative degradation of polymer chains; the EC3.5.1.4 amidase (encoded by L6_GM002044/L6_GM002722) implicated in acrylamide degradation; and the EC2.3.1.16 acyltransferase (L6_GM005190), associated with ethylbenzene degradation.

Additionally, a comprehensive safety assessment is essential. On one hand, the potential ecological or toxicological effects of the transformation products remain to be evaluated. On the other hand, given the characteristic of the *Bacillus cereus* strain used as an opportunistic pathogen, it is imperative to strictly prevent the ecological and health risks associated with the release of live bacteria in any practical application. Feasible strategies include extracting and purifying the degradative enzyme systems for direct use, thereby avoiding the dispersal of live cells [59], or employing genetic engineering to knock out virulence-related genes, thereby constructing engineered strains with high degradation efficiency and no pathogenicity [60,61]. Future efforts should integrate strategies and rigorous risk assessment, to enable the safe and effective translation of this technology.

## 4. Conclusions

A strain L6, taxonomically assigned by barcode region as *Bacillus cereus*, was screened from a soil covered with long-term agricultural films and capable of inducing the biodeterioration of both LDPE- and PBAT-MPs. A comprehensive suite of characterization techniques revealed that this process entails pitting, increased roughness, decreased hydrophobicity, crystallinity, thermal stability, and changed the functional groups and chemical structure. MPs biodeterioration in the presence of strain L6 could be attributed to its ability to form a biofilm, and to secrete laccase and oxygenase to catalyze the redox reaction. In addition, genomic analysis further identified chromosomal and plasmid-borne predicted genes encoding putative enzymes for polymer and additive biodeterioration. These results establish strain L6 as a promising microbial candidate for the bioremediation of mixed plastic pollution. This study provides a candidate strain for the biodeterioration of MPs, which may be further improved, or which may be combined with other methods, contributing to the use of microorganisms in mitigating the contamination of MPs.

## Data Availability

The original contributions presented in this study are included in the article/Supplementary Material. Further inquiries can be directed to the corresponding author.

## Supplementary Materials

The following supporting information can be downloaded at: https://www.mdpi.com/article/10.3390/microorganisms14010179/s1, Text S1: The specific formulations of medium; Text S2: Information on primer selection; Text S3: Detailed information on instrument operating conditions for LDPE- and PBAT-MPs characterization; Text S4: Detailed methods of whole genome sequencing and annotation; Table S1: Effects of different MPs groups on weight loss and WCAs based on a One-way ANOVA analysis; Table S2: Three pathways associated with biofilm formation and annotated genes in strain L6; Table S3: Results of repeated measures ANOVA on OD600 values with different MPs groups as the independent variables and time as the repeated measure; Table S4: Elemental composition of LDPE and PBAT MPs; Table S5: CAZy family and annotated genes in strain L6; Table S6: KEGG metabolic pathway and related genes and enzymes; Figure S1: Statistical map of CAZy functional classification and number of corresponding genes. CBM: number of genes for carbohydrate-related modules (CBMs). CE: number of genes for glycoside esterases (CEs). GH: number of genes for glycoside hydrolases (GHs). GT: number of genes for glycosyltransferases (GTs). PL: number of genes for polysaccharide lyases (PLs). AA: number of genes for oxidoreductases (AAs); Figure S2: Styrene degradation metabolic pathway of strain L6 from the KEGG database. Red highlights indicate the presence of functional enzymes involved in metabolism; Figure S3: Ethylbenzene degradation metabolic pathway of strain L6 from the KEGG database. Red highlights indicate the presence of functional enzymes involved in metabolism; Figure S4: Xylene degradation metabolic pathway of strain L6 from the KEGG database. Red highlights indicate the presence of functional enzymes involved in metabolism; Figure S5: Limonene and pinene degradation metabolic pathway of strain L6 from the KEGG database. Red highlights indicate the presence of functional enzymes involved in metabolism; Figure S6: Naphthalene degradation metabolic pathway of strain L6 from the KEGG database. Red highlights indicate the presence of functional enzymes involved in metabolism. Reference [62] is cited in the Supplementary Materials.
