# Supplementary material for: Kinetics and Potential Mechanisms of LDPE and PBAT Microplastics Biodeterioration by Soil Bacteria Bacillus cereus L6
Source: Microorganisms. 2026 Jan 14;14(1):179. doi: 10.3390/microorganisms14010179 (PMC12844225; doi:10.3390/microorganisms14010179)
Supplement: Supplementary file 1 [file microorganisms-14-00179-s001.zip › microorganisms-4043184-supplementary.pdf]

Supplementary materials for

LDPE and PBAT Microplastics Degradation Kinetics and Mechanisms in the presence of Soil Bacteria Bacillus cereus L6

Jiayang Hu[1,2], Tianyu Liu[2,3], Jinpeng Zhang[2], Yong Yu[2], Jincai Ma[1], Yanjun Li [2,*]

[1] Key Laboratory of Ground Water Resource and Environment, Ministry of Education, Jilin University, Changchun 130021, China

[2] Jilin Provincial Key Laboratory of Environmental Ecology in Black Soils, Research Center of Regional Development and Environment, Northeast Institute of Geography and Agroecology, Chinese Academy of Sciences, Changchun, 130102, China

[3] College of Horticulture, Jilin Agricultural University, Changchun 130118, China

***Corresponding author:** Yanjun Li

E-mail: liyanjun@iga.ac.cn

Text S1. The specific formulations of medium

Text S2. Information on primer selection.

Text S3. Detailed information on instrument operating conditions for LDPE- and PBAT-MPs characterization.

Text S4. Detailed methods of whole genome sequencing and annotation.

Table S1. Effects of different MPs groups on weight loss and WCAs based on a One-way ANOVA analysis.

Table S2. Three pathways associated with biofilm formation and annotated genes in strain L6.

Table S3. Results of repeated measures ANOVA on OD600 values with different MPs groups as the independent variables and time as the repeated measure.

Table S4. Elemental composition of LDPE and PBAT MPs

Table S5. CAZy family and annotated genes in strain L6.

Table S6. KEGG metabolic pathway and related genes and enzymes.

Fig. S1. Statistical map of CAZy functional classification and number of corresponding genes. CBM: number of genes for carbohydrate-related modules (CBMs). CE: number of genes for glycoside esterases (CEs). GH: number of genes for glycoside hydrolases (GHs). GT: number of genes for glycosyltransferases (GTs). PL: number of genes for polysaccharide lyases (PLs). AA: number of genes for oxidoreductases (AAs).

Fig. S2. Styrene degradation metabolic pathway of strain L6 from the KEGG database. Red highlights indicate the presence of functional enzymes involved in metabolism.

Fig. S3. Ethylbenzene degradation metabolic pathway of strain L6 from the KEGG database. Red highlights indicate the presence of functional enzymes involved in metabolism.

Fig. S4. Xylene degradation metabolic pathway of strain L6 from the KEGG database. Red highlights indicate the presence of functional enzymes involved in metabolism.

Fig. S5. Limonene and pinene degradation metabolic pathway of strain L6 from the KEGG database. Red highlights indicate the presence of functional enzymes involved in metabolism.

Fig. S6. Naphthalene degradation metabolic pathway of strain L6 from the KEGG database. Red highlights indicate the presence of functional enzymes involved in metabolism.

Text S1. The specific formulations of medium

The specific formulations of medium are as follow [1].

Inorganic Salt Medium ($g\cdot L^{-1}$): 0.92 g $K_2HPO_4\cdot 3H_2O$, 0.7 g KH_2PO_4, 0.7 g $MgSO_4\cdot 7H_2O$, 1.0 g NH_4NO_3, 0.005 g NaCl, 0.002 g $FeSO_4\cdot 7H_2O$, 0.002 g $ZnSO_4\cdot 7H_2O$, 0.001 g $MnSO_4\cdot H_2O$, pH ≈ 7.2; For solid medium, add 18–20 g agar powder.

Trace Carbon Source Medium ($g\cdot L^{-1}$): 0.5 g yeast extract, 2.0 g $(NH_4)_2SO_4$, 100 mL trace element solution; For solid medium, add 18–20 g agar powder.

Trace Element Stock Solution ($g\cdot L^{-1}$): 1.0 g $FeSO_4\cdot 7H_2O$, 1.0 g $MgSO_4\cdot 7H_2O$, 0.1 g $CuSO_4\cdot 5H_2O$, 0.1 g $MnSO_4\cdot H_2O$, 0.1 g $ZnSO_4\cdot 7H_2O$, pH ≈ 7.0.

LB Nutrient Medium ($g\cdot L^{-1}$): 10 g peptone, 3 g beef extract, 5 g NaCl, pH = 7.2; For solid medium, add 18–20 g agar powder.

Text S2. Information on primer selection

Primers 27F (5'-AGAGTTTGATCCTGGCTCAG-3') and 1492R (5'-GGTTACCTTGTTACGACTT-3') were selected for polymerase chain reaction (PCR) amplification of strain L6. PCR products were screened and purified by agarose gel electrophoresis and sent to Bioengineering Biotech (Shanghai, China) Co. for sequencing.

Text S3. Detailed information on instrument operating conditions for LDPE- and PBAT-MPs characterization.

A scanning electron microscope (JSM-IT500, Japan) was used to observe the changes in surface morphology of LDPE- and PBAT-MPs after 28 d of incubation in strain L6. For this purpose, pristine and degraded LDPE- and PBAT-MPs were sputter-plated with a pt layer of 25 mA in an argon atmosphere at 0.3 MPa, and then observed at 5000× magnification (for surface morphology observation), including bacterial colonisation of the microplastic surface.

EDS was conducted along with SEM analysis. The L6-treated and control LDPE- and PBAT-MPs without bacterial adhesion were used for EDS analysis. It involves the passing of high-energy X-ray beams on the plastic film.

To compare surface erosions on the outer layer of both control and L6-treated LDPE- and PBAT-MPs, the AFM instrument, WITEC alpha 300 A (tapping mode), was used. The LDPE- and PBAT-MPs were sterilized with 2% SDS solution, 70% ethanol and distilled water.

Surface sterilized LDPE- and PBAT-MPs (both control and L6-treated MPs) were taken, and the surface contact angle was measured at room temperature by placing a drop of water on it. In this experiment, the images of the water contact angle were captured within 10 s using Data Physics GmbH contact angle instrument, Germany (model OCA 15 plus video-based optical instrument with SCA20 software system) and recorded the images for comparison studies.

Changes in functional groups for LDPE- and PBAT-MPs were analyzed by Fourier Transform Infrared Spectroscopy (FTIR). The MP samples were mixed with dried potassium bromide in a ratio of 1:100 and compressed into flakes for testing. Infrared spectra were recorded in the 4000-400 cm^{-1} region with a resolution of 4 cm^{-1} and the number of scans was 64.

The XRD analyses were carried out without grinding the samples, and the LDPE- and PBAT-MPs were directly taken and added to the middle of the grooves of the sample frame. The crystallinity of MPs was calculated using MDI Jade (Version 6.5). The data are imported into Jade, the full spectrum is fitted to the diffraction peaks within

the test range, and the pseudo-Voigt function is used to describe the diffraction peaks. The Jade software gives the crystallinity value based on the fitted values of the integrated area of the crystalline and amorphous peaks

The TGA was performed on both the control and L6-treated LDPE- and PBAT-MPs utilizing the Perkin Elmer TGA 4000 thermogravimetric analyzer. The analysis involved subjecting the samples to a controlled heating process, from room temperature and to up to 600◦C. The heating rate was set at 10◦C min^{-1}, and a constant nitrogen flow of 10 mL/min was maintained throughout the experiment.

Text S4. Detailed methods of whole genome sequencing and annotation.

Genome sequencing and assembly

Genomic DNA was extracted with the STE method. The harvested DNA was detected by the agarose gel electrophoresis and quantified by Qubit. The genome of L6 was sequenced by Single Molecule, Real-Time (SMRT) technology. Sequencing was performed at the Beijing Novogene Bioinformatics Technology Co., Ltd. The low quality reads were filtered by the SMRT Link v8.0 and the filtered reads were assemblied using software Canu to generate one contig without gaps. Canu:(https://github.com/marbl/canu/,version: 2.0)

Genome Component prediction

Genome component prediction included the prediction of the coding gene, repetitive sequences, non-coding RNA, genomics islands, transposon, prophage, and clustered regularly interspaced short palindromic repeat sequences (CRISPR). The available steps were proceeded as follows: 1) For bacteria, we used the GeneMarkSprogram to retrieve the related coding gene. 2) The interspersed repetitive sequences were predicted using the RepeatMasker (http://www.repeatmasker.org/). The tandem Repeats were analyzed by the TRF (Tandem repeats finder). 3) Transfer RNA (tRNA) genes were predicted by the tRNAscan-SE. Ribosome RNA (rRNA) genes were analyzed by the rRNAmmer. Small nuclear RNAs(snRNA)were predicted by BLAST against the Rfam database. 4) The IslandPath-DIOMB program was used to predict the Genomics Islands, and transposon PSI was used to predict the transposons based on the homologous blast method. The PHAST was used for the prophage prediction (http://phast.wishartlab.com/) and the CRISPRFinder was used for the CRISPR identification.

Gene function

We used seven databases to predict gene functions. They were respective GO (Gene Ontology), KEGG (Kyoto Encyclopedia of Genes and Genomes), COG (Clusters of Orthologous Groups), NR (Non-Redundant Protein Database databases), TCDB

(Transporter Classification Database), and, Swiss-Prot. A whole genome Blast search (E-value less than 1e^{-5}, minimal alignment length percentage larger than 40%) was performed against above seven databases. The secretory proteins were predicted by the Signal P database, and the prediction of Type I-VII proteins secreted by the pathogenic bacteria were based on the EffectiveT3 software. Meanwhile, we analyzed the secondary metabolism gene clusters by the antiSMASH.

Comparative genomics analysis

Comparative genomic analysis included the genomic synteny, the core genes and specific genes, gene family phylogenetic tree, SNP (Single Nucleotide Polymorphism), indel (insertion and deletion) and SV (Structural Variation) annotation, and genome visualization. 1) Genomic alignment between the sample genome and reference genome (or among more than two sample genomes) were performed using the MUMmer and LASTZ tools. Genomic synteny was analyzed based on the alignment results. 2) Core genes and specific genes were analyzed by the CD-HIT rapid clustering of similar proteins software with a threshold of 50% pairwise identity and 0.7 length difference cutoff in amino acid. Then the venn figure was drawn to show their relationships among the samples. 3) Blast was used to pairwise align all genes and eliminate the redundancy by solar and carried out gene family clustering treatment based on the alignment results with Hcluster_sg software. 4) The phylogenetic tree was constructed by the TreeBeST or PhyML and the setting of bootstraps was 1,000 with the orthologous genes. 5) SNP, indel and SV were found by the genomic alignment results among samples by the MUMmer and LASTZ we mentioned them before. 6) Genome overview was created by Circos to show the annotation information.

Table S1. Effects of different MPs groups on weight loss and WCAs based on a One-way ANOVA analysis.

Parameters	F	p
Weight loss	79.561	<0.001
WCAs	23.553	<0.001

Table S2. Three pathways associated with biofilm formation and annotated genes in strain L6.

Pathway ID	Pathway	Genes
map02026	Biofilm formation-*Escherichia coli*	L6_GM004994, L6_GM005066, L6_GM005067, L6_GM005068, L6_GM005069, L6_GM005485
map02025	Biofilm formation-*Pseudomonas aeruginosa*	L6_GM001444, L6_GM001445
map05111	Biofilm formation-*Vibrio cholerae*	L6_GM000302, L6_GM001820, L6_GM003003, L6_GM003876, L6_GM004994, L6_GM005291, L6_GM005360, L6_GM005485, L6_GM005584, L6_GM000153

Table S3. Results of repeated measures ANOVA on OD600 values with different MPs groups as the independent variables and time as the repeated measure.

	F	P
Groups	387.914	<0.001
Time	34.637	<0.001
Group×Time	14.064	<0.001

Table S4. Elemental composition of LDPE and PBAT MPs

Element analysis of PBAT-MPs treated with L6

Element	Type of Line	Weight %	Atomic%
C	K	57.21±0.05	64.04±0.06
O	K	42.79±0.09	35.96±0.08
Total		100.00	100.00

Element analysis of control PBAT-MPs

Element	Type of Line	Weight %	Atomic%
C	K	77.12±0.06	81.79±0.06
O	K	22.88±0.08	18.21±0.06
Total		100.00	100.00

Element analysis of untreated PBAT-MPs

Element	Type of Line	Weight %	Atomic%
C	K	79.97±0.06	84.17±0.06
O	K	20.03±0.08	15.83±0.06
Total		100.00	100.00

Element analysis of untreated LDPE-MPs

Element	Type of Line	Weight %	Atomic%
C	K	100.00±0.07	100.00±0.07
Total		100.00	100.00

Element analysis of control LDPE-MPs

Element	Type of Line	Weight %	Atomic%
C	K	100.00±0.09	100.00±0.09
Total		100.00	100.00

Element analysis of LDPE-MPs treated with L6

Element	Type of Line	Weight %	Atomic%
C	K	93.37±0.06	94.94±0.06
O	K	6.63±0.05	5.06±0.04
Total		100.00	100.00

Table S5. CAZy family and annotated genes in strain L6.

CAZy family	Match number	Genes
AA	5	L6_GM000271, L6_GM002922, L6_GM002956, L6_GM005876, L6_GM005908

Table S6. KEGG metabolic pathway and related genes and enzymes.

Pathway ID	Pathway	Genes	Enzyme
map00643	Styrene degradation	L6_GM002044, L6_GM002722	EC3.5.1.4
map00642	Ethylbenzene degradation	L6_GM005190	EC2.3.1.16
map00622	Xylene degradation	L6_GM003690, L6_GM004562, L6_GM005543	EC 1.13.11.2, EC5.3.2.6
map00903	Limonene and pinene degradation	L6_GM001485, L6_GM002962, L6_GM003713	EC1.2.1.3
map00626	naphthalene degradation	L6_GM005698	EC1.1.1.1

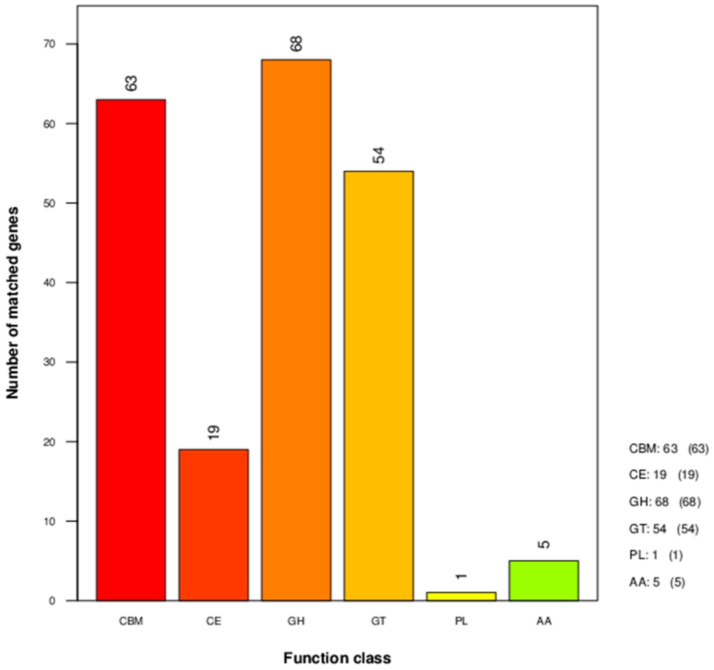

Fig. S1. Statistical map of CAZy functional classification and number of corresponding genes. CBM: number of genes for carbohydrate-related modules (CBMs). CE: number of genes for glycoside esterases (CEs). GH: number of genes for glycoside hydrolases (GHs). GT: number of genes for glycosyltransferases (GTs). PL: number of genes for polysaccharide lyases (PLs). AA: number of genes for oxidoreductases (AAs).

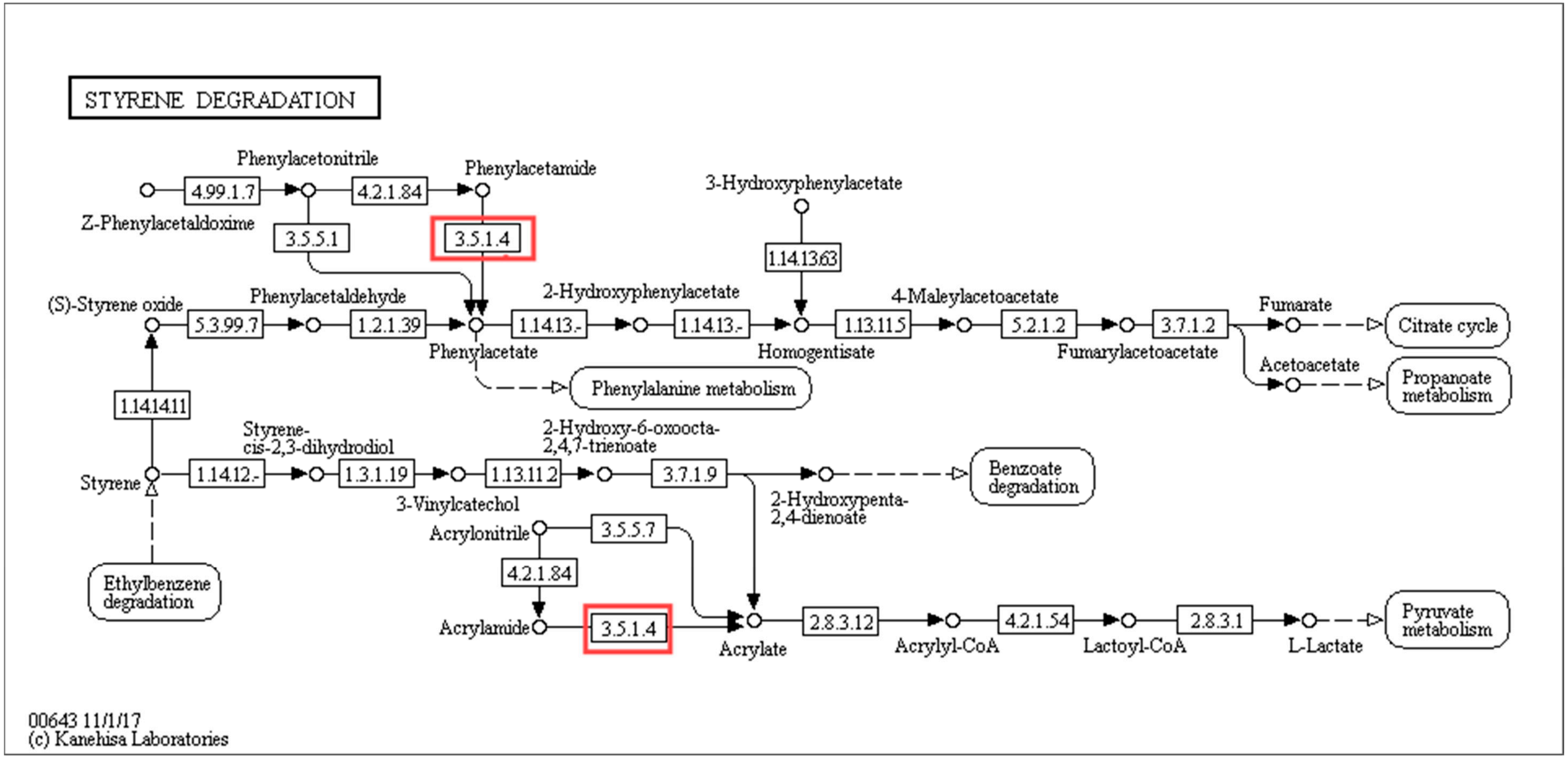

Fig. S2. Styrene degradation metabolic pathway of strain L6 from the KEGG database. Red highlights indicate the presence of functional enzymes involved in metabolism.

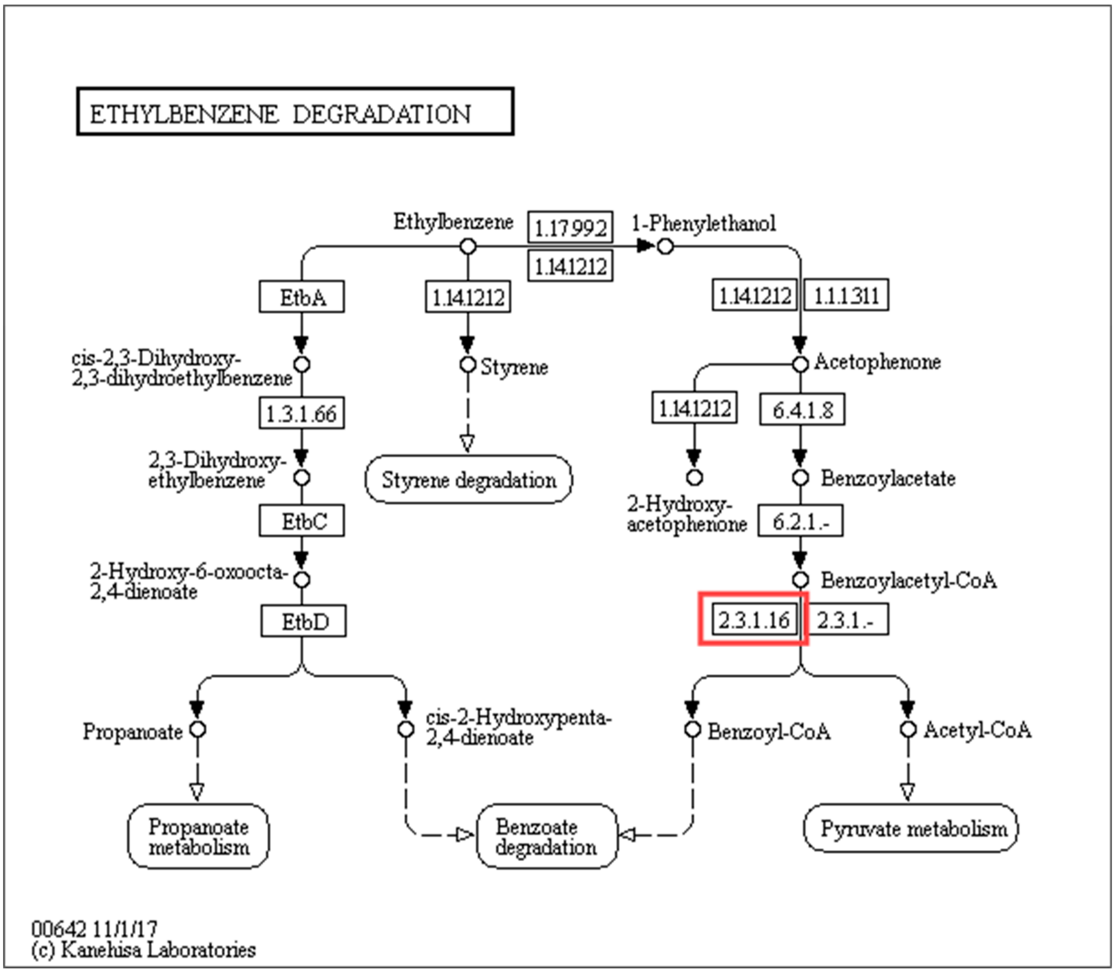

Fig. S3. Ethylbenzene degradation metabolic pathway of strain L6 from the KEGG database. Red highlights indicate the presence of functional enzymes involved in metabolism.

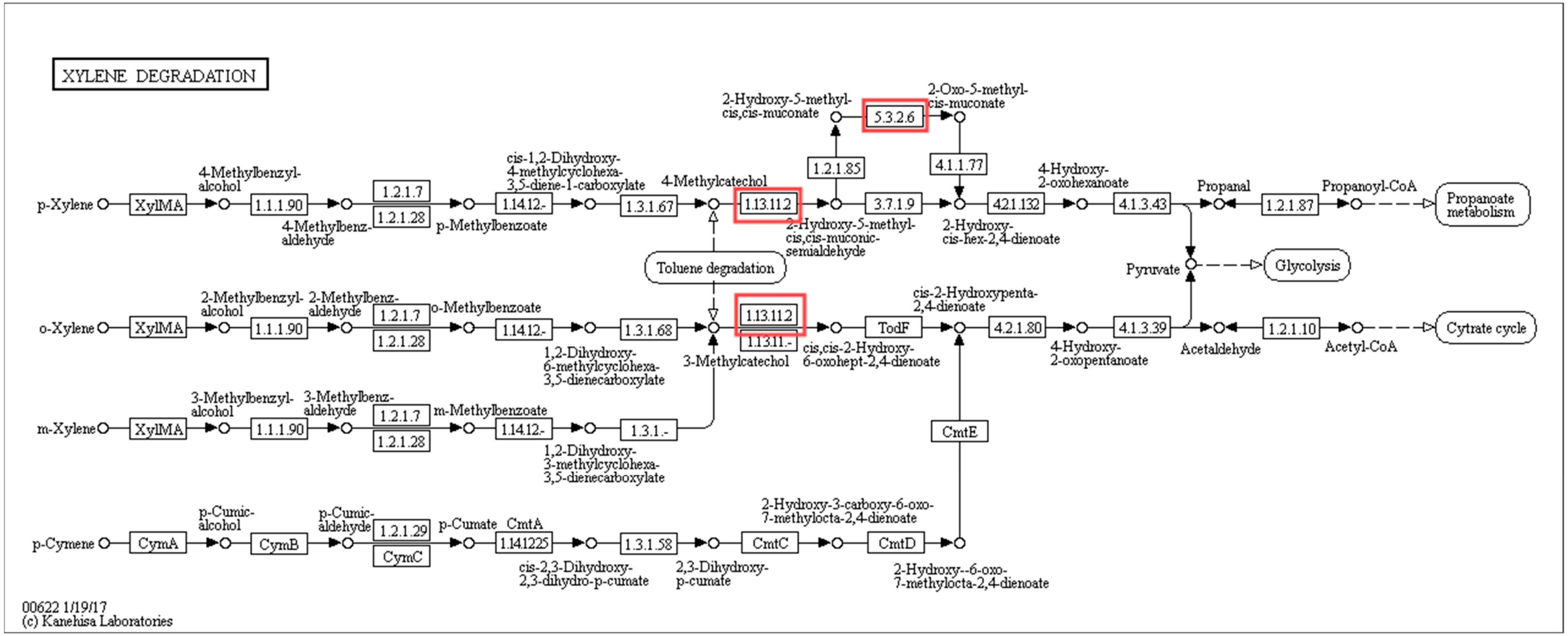

Fig. S4. Xylene degradation metabolic pathway of strain L6 from the KEGG database. Red highlights indicate the presence of functional enzymes involved in metabolism.

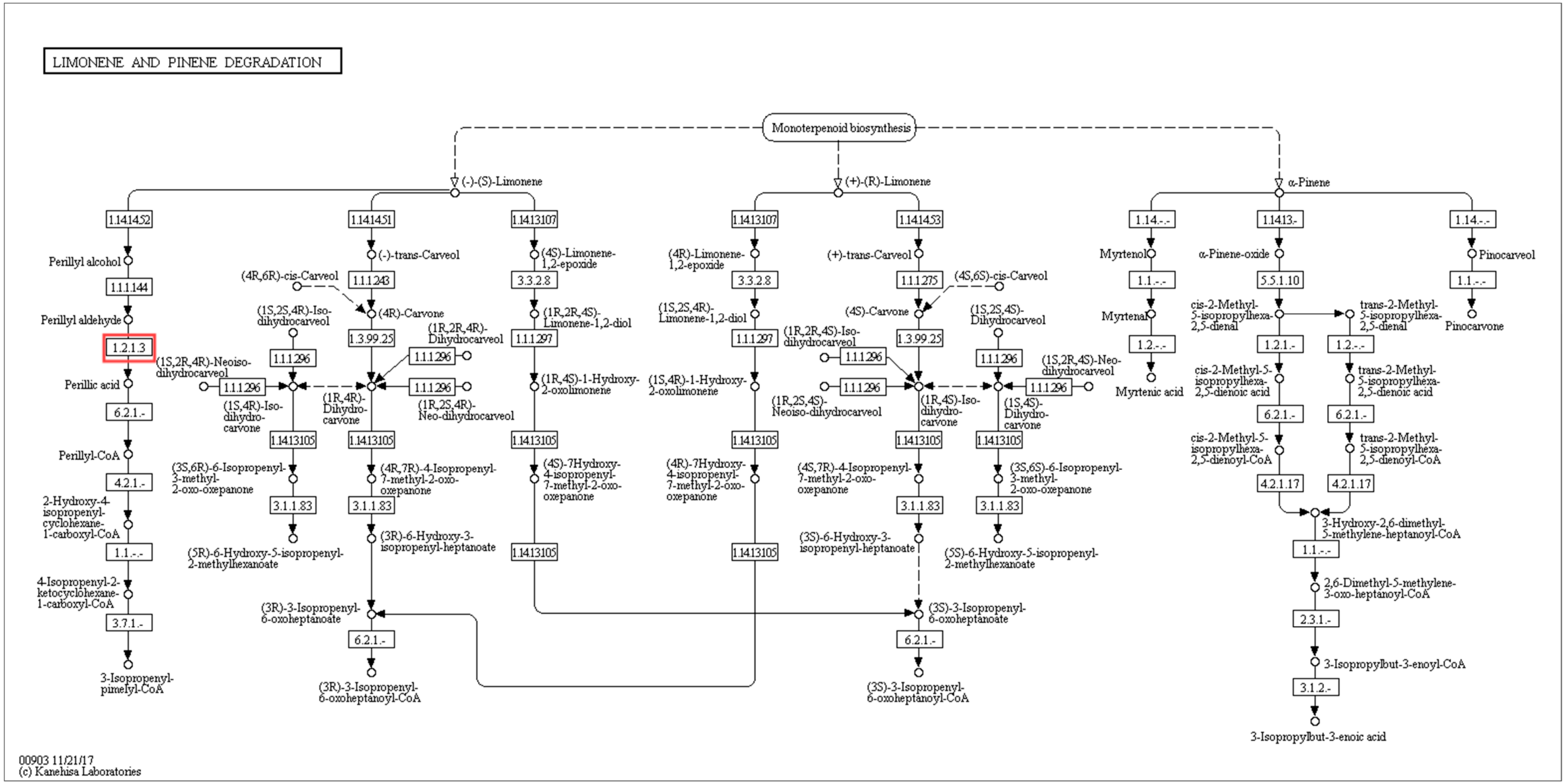

Fig. S5. Limonene and pinene degradation metabolic pathway of strain L6 from the KEGG database. Red highlights indicate the presence of functional enzymes involved in metabolism.

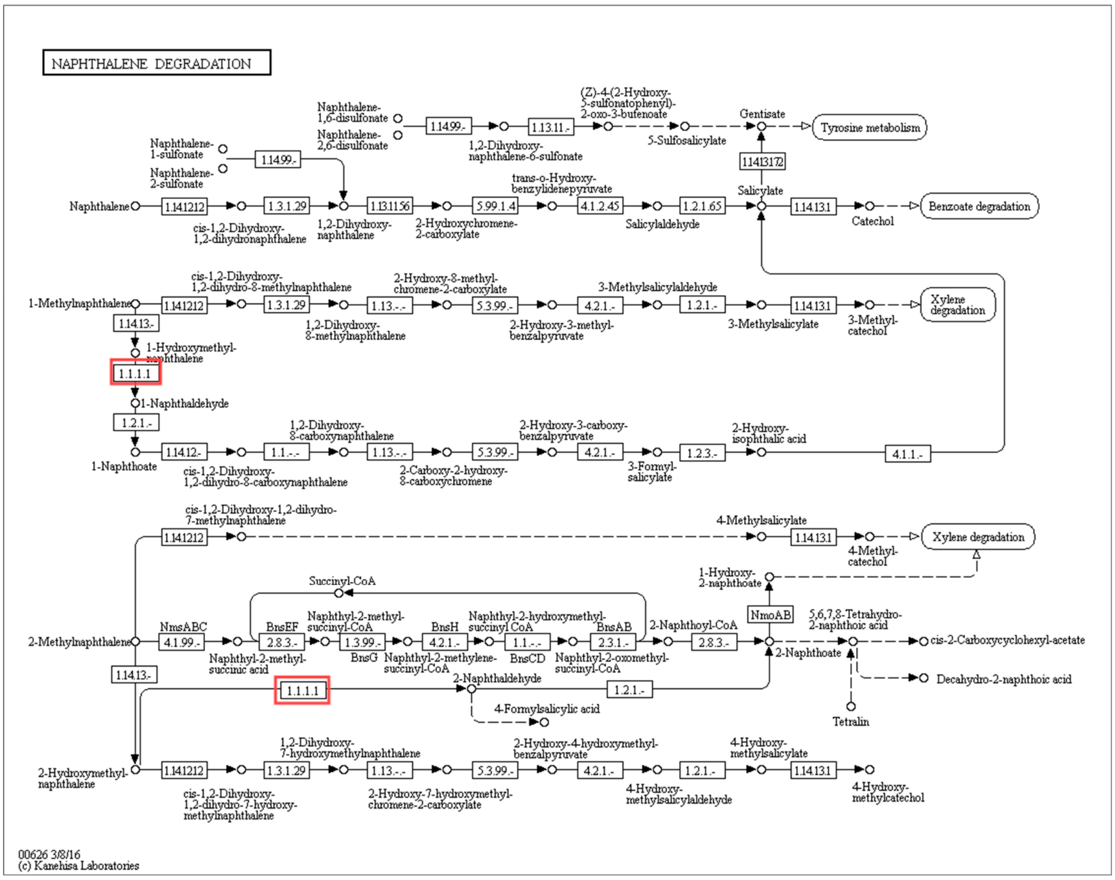

Fig. S6. Naphthalene degradation metabolic pathway of strain L6 from the KEGG database. Red highlights indicate the presence of functional enzymes involved in metabolism.

Reference:

[1] Hou, L. Screening of Plastic-Degrading Microorganisms for Different Polymer Types and Preliminary Investigation of Their Degradation Mechanisms. Master, Northwest Agriculture and Forestry University, 2020.
